# Modeling In Vitro Biofilm–Calculus Formation for Assessing Periodontal Instrumentation and the Forces Applied

**DOI:** 10.1002/cre2.70359

**Published:** 2026-04-22

**Authors:** Gert Jungbauer, Luca Giacobbo, Alexandra Stähli, Anton Sculean, Jürgen Burger, Sigrun Eick, Martin Hofmann

**Affiliations:** ^1^ Department of Periodontology, School of Dental Medicine University of Bern Bern Switzerland; ^2^ School of Biomedical and Precision Engineering University of Bern Bern Switzerland

**Keywords:** dental biofilm and calculus, force evaluation, manual and piezoelectric ultrasonic scaler, periodontal therapy

## Abstract

**Objectives:**

In vitro models provide valuable insights into treatment options and their effectiveness prior to and alongside clinical evaluation. Such models should be standardized, reproducible, and closely reflect the clinical situation. This study aimed to investigate the removal of subgingival biofilm and calculus by instrumentation, which is vital in the successful treatment of periodontitis. The approach was to (i) develop an in vitro model based on biofilm and calculus formation and (ii) assess it by hand and ultrasonic instrumentation, while (iii) measuring the forces applied in an innovative periodontal defect model.

**Materials and Methods:**

A multi‐species mixture consisting of 11 bacterial strains was used to form an initial calculus over 14 days. Inserts carrying human dentin specimens, either with biofilm or with a combination of biofilm and calculus, were placed in a periodontal pocket model equipped with a multi‐axis force sensor, followed by treatment with hand or ultrasonic instrumentation. Instrumentation forces were recorded, and the remaining biofilm or biofilm/calculus was analyzed for bacterial colony‐forming unit (cfu) counts and calcium levels after instrumentation.

**Results:**

The results revealed that the cfu counts and calcium levels in the biofilm/calculus group were higher compared to the respective biofilm controls. Ultrasonic instrumentation was more effective than hand instrumentation in reducing cfu counts in both the biofilm and biofilm/calculus groups. Furthermore, both hand and ultrasonic instrumentation reduced calcium levels in the biofilm/calculus groups. The peak forces Fy in the hand instrumentation groups were significantly higher in both the biofilm and biofilm/calculus groups compared to the respective ultrasonic groups.

**Conclusions:**

The model enabled an initial reproducible calculus formation and evaluation of different instrumentation modalities, including the forces applied. The results favored the ultrasonic instrumentation due to its superior removal of biofilm and calculus and lower lateral forces. The presented biofilm/calculus model offers a new in vitro approach for comparing different instrumentation modalities.

## 1 Introduction

Before new treatment methods are introduced in clinical practice, they are typically validated and optimized using in vitro and animal models. However, due to ethical and socioeconomic concerns, there is strong motivation to replace animal models with modern in vitro alternatives (Clift and Doak 2021). Biofilm models mimic the three‐dimensional structure and integrate microorganisms relevant to the respective disease. Furthermore, the influence of temperature, shear flow, nutrient and oxygen supply, microbial metabolism, the composition of the extracellular matrix, and mechanical properties is also incorporated (Crivello et al. 2023). Models of varying complexity are used to evaluate therapeutic approaches. Although these models are all suitable for assessment, translating the findings into clinical practice may result in reduced treatment efficacy due to the increased complexity of in vivo conditions (Eick 2021). Therefore, a periodontal pocket model was developed to enable comparison of different mechanical approaches for biofilm debridement (Hägi et al. 2015).

Dental biofilm is a multi‐species microbial community embedded in an extracellular matrix (Karygianni et al. 2020). Periodontitis is a biofilm‐induced and host‐mediated disease characterized by the progressive degradation of the tooth‐supporting tissue. Bacteria commonly found in the dysbiotic biofilm in periodontitis include *Tannerella forsythia*, *Porphyromonas gingivalis*, *Treponema denticola*, and *Fusobacterium nucleatum*, among others (Yekani et al. 2025).

The prevalence of periodontitis in dentate individuals has been estimated at 61.6%, with age being a crucial confounding factor (Trindade et al. 2023). Periodontitis is associated with many systemic diseases such as cardiovascular disease, Type 2 diabetes, pneumonia, Alzheimer's disease, rheumatoid arthritis, and cancer. The inflammation associated with periodontitis may contribute to this link, and vice versa, the systemic condition might increase the periodontal inflammation (Hajishengallis 2022).

The biofilm may serve as a matrix for calculus formation; a locally increased pH enables the precipitation of calcium phosphate crystals. Supra‐ and subgingival calculus differ in color, crystalline structure, mineral content, and incorporated microorganisms (Roberts‐Harry and Clerehugh 2000). The calcification rate is highly individual and depends on various factors, such as dietary sugars, salivary content, age, ethnicity, gender, and bacterial load (Akcalı and Lang 2018). The mineralization seems to be completed within 12 days (Akcalı and Lang 2018; Clift and Doak 2021). Subgingival calculus itself does not appear to have any pathogenic potential but acts as a reservoir for viable periopathobionts (Calabrese et al. 2007). This might be confirmed by a clinical study, where the presence of subgingival calculus was associated with progressive attachment loss within 5 years in adolescents (Clerehugh et al. 1995). In an animal model, periodontitis was induced by transferring human calculus from periodontitis patients to subgingival sites in rats (You et al. 2025). To analyze dental calculus removal, either extracted teeth with calculus (Lampe Bless et al. 2011), calculus models based on cyanoacrylate (Sirinirund et al. 2019), or composite materials (Stutzer et al. 2022, 2024) are used. To the best of our knowledge, a combined multi‐species biofilm–calculus model has not yet been applied to an in vitro assay.

According to the guidelines set by the European Federation of Periodontology, the elimination of subgingival biofilm and calculus is crucial for the successful treatment of periodontitis, which can be performed using hand and/or ultrasonic scalers (Sanz et al. 2020). The instrumentation should efficiently remove the deposits while avoiding damage to the tooth surface. The unwanted defects, such as tooth substance loss and increased surface roughness, depend significantly on the force applied (Arabaci et al. 2007; Flemmig et al. 1998; Lampe Bless et al. 2011; Lea et al. 2009b). In addition, excessive force reduces the efficiency of ultrasonic scalers by decreasing the displacement amplitude and may cause elliptical oscillating patterns (Lea et al. 2009a; Stutzer et al. 2024). Flemming et al. recommended using tip angulation of close to 0° and limiting the lateral force applied to the tooth surface during ultrasonic treatment to 0.5 N, a measure that complies with manufacturers' recommendations (Flemmig et al. 1998). In general, force measurements during periodontal instrumentation have been reported very rarely and are often limited by a lack of information on force directionality (Ruppert et al. 2002; Zappa et al. 1991) or by the use of artificial calculus models (Stutzer et al. 2024). To date, reproducible and detailed force measurements using an in vitro pocket model incorporating biofilm and calculus, allowing for a standardized and realistic comparison of different instrumentation modalities, have not been carried out.

The aim of this study was (i) to establish an in vitro model based on biofilm and calculus formation, (ii) to assess the reduction of the in vitro biofilm and calculus by hand and ultrasonic instrumentation, while (iii) measuring the forces applied in an innovative periodontal defect model.

## 2 Materials and Methods

### 2.1 Pocket Model

**Figure 1:**
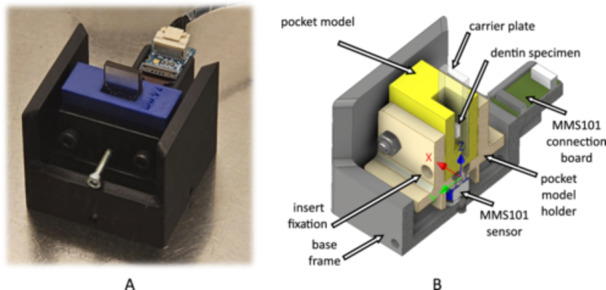
Periodontal pocket model capable of measuring three‐dimensional forces with (A) fixed insert carrying a dentin specimen; (B) cross‐section of pocket model, force sensor, and holder.

To simulate the clinical situation, a periodontal pocket model, as already described (Berto et al. 2024; Hägi et al. 2015), was modified and improved to allow measurements of the applied forces during instrumentation (Figure 1). For this purpose, the pocket model was equipped with a 6‐axis force–torque sensor (MMS101, Mitsumi Electric Co., Tokyo, Japan) with an effective resolution of 0.04–0.06 N, depending on the orientation, in a range of up to ± 40 N. The sensor data was acquired at the sensor's sampling rate of 50 Hz using a MATLAB script (MathWorks, Massachusetts, USA) on an external computer. Both the pocket model and its holder, which shields the sensor from splashing water, were manufactured using 3D printing with fused deposition modeling (X1E, Bambu Lab, Shenzhen, China) from ABS.

Inserts carrying human dentin specimens (with biofilm and in part with calculus) were placed in the pocket model and fixed with an M4 screw. For specimen preparation, extracted human teeth were obtained from volunteers with written consent, after being informed about their use in research. Ethical approval was not required in accordance with the guidelines of the Cantonal Ethical Committee Bern (KEK), as the teeth were categorized as “irreversibly anonymized.” The dentin slices were trimmed and smoothed to a size of 5 mm × 5 mm × 1 mm (length × width × height) using diamond burs and sandpaper. The dentin specimens were bonded to polycarbonate carrier plates using cyanoacrylate and autoclaved at 121°C for 20 min.

### 2.2 In Vitro Calculus and Biofilm Formation

In preliminary experiments, the calcifying ability of selected bacterial strains was assessed. Supragingival and subgingival calculus samples were taken and forwarded to cultivation with 10% CO_2_ and in an anaerobic atmosphere. The protocol was approved by the ethical commission of the University of Regensburg, Germany (25‐4064‐101, 03/03/2025). Isolated strains, as well as reference strains, were cultured for 2 weeks using a protocol described by Sidaway (1978) and Sissons et al. (1991), with a minor modification: brain–heart infusion (BHI) broth was used instead of water. Calcification was analyzed by von Kossa staining (Sattary et al. 2019; Sidaway 1979). Thereafter, four mixtures were created from the selected strains and assessed for their calcifying potential by the same protocol. Finally, the multi‐species mixture consisted of 11 bacterial strains:

1. *Streptococcus gordonii* Be‐C9/32
2. *Streptococcus mitis* Be‐C9/24B
3. *Streptococcus oralis* Be‐9/26
4. *Streptococcus sanguinis* Be‐9/11
5. *Streptococcus constellatus* Be‐9/25
6. *Rothia dentocariosa* Be‐9/27B
7. *Actinomyces gerencseriae* Be‐C9/30
8. *Cardiobacterium valvarum* 9/18
9. *F. nucleatum* ATCC 25586
10. *T. forsythia* ATCC 43037
11. *P. gingivalis* ATCC 33277

The clinical isolates (Strains 1–8) were identified by MALDI‐TOF.

After performing several optimization steps, the protocol for biofilm/calculus formation was as follows: The strains were precultured on tryptic soy agar (TSA) plates (Oxoid, Basingstoke, UK) with 5% sheep blood with 10% CO_2_ or anaerobically (Strains 9–11). Strains 1–8 (bacterial Mixture A) were suspended in 0.9% w/v NaCl according to optical density OD_600_ = 0.2 (equivalent to 1.6 × 10^8^ bacteria/mL) each, Strains 9–11 (bacterial Mixture B) according to OD_600_ = 1 (equivalent to 8 × 10^8^ bacteria/mL). For bacterial Mixture A, each one part of strain suspensions 1–5 and each four parts of the Strains 6–8 were mixed and added to BHI broth supplemented with 1 ppm sodium fluoride in a ratio of 1:9. For bacterial mixture B, each one part of the strain Suspensions 9–11 was mixed and added in a ratio of 1:9 to BHI broth with 5 mg/L hemin and 10 mg/L *N*‐acetylmuramic acid (Sigma‐Aldrich/Merck, Darmstadt, Germany).

**Figure 2:**
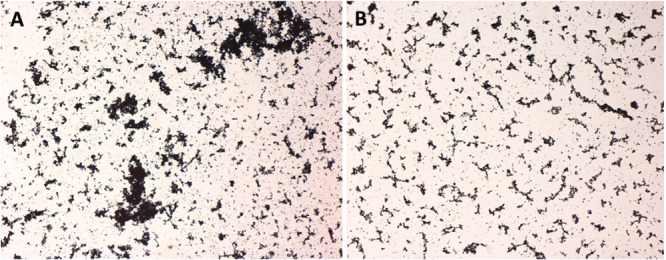
Calcification visualized by von Kossa staining in biofilm/calculus (A) and biofilm only (B) cultivation.

**Figure 3:**
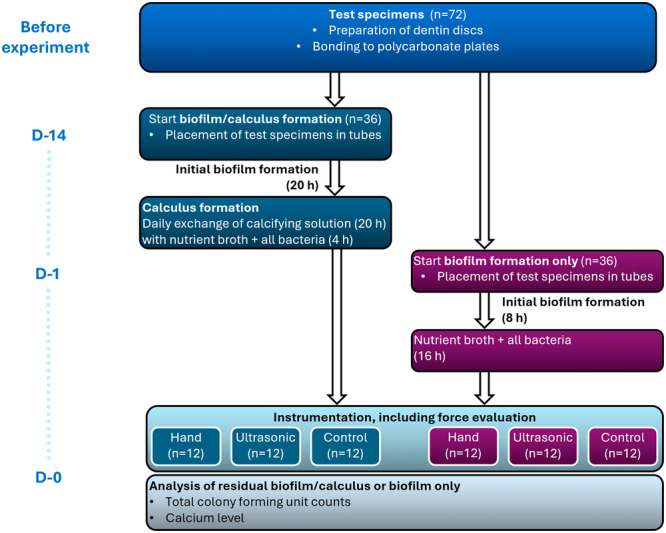
Flowchart of biofilm only and combined biofilm/calculus formation, as well as sample allocation for untreated control, hand instrumentation, and ultrasonic instrumentation.

The in vitro calculus formation was conducted over a course of 14 days. The dentin specimens were coated with 50 µL of a 0.67% mucin/1.5% bovine serum albumin solution for 1 h (pellicle formation). Thereafter, the specimens were transferred into polystyrene tubes. For the initial biofilm formation, 2.5 mL of bacterial Mixture A was added and incubated for 20 h at 10% CO_2_. Then, the culture medium was carefully removed and replaced with 2.5 mL calcifying solution prepared according to Sidaway (1978) and adjusted to a pH of 7.5. (The calcifying solution contained 2.1 g NaCl, 1.56 g KCl, 0.9 g Na_2_HPO4·12H_2_O, 0.39 g NaH_2_PO4· 2H_2_O, 0.87 g KSCN 0.87 g, 0.2 g urea, 0.8 g 3,3‐dimethylglutaric acid, 0.36 g NaOH, and 0.33 g CaCl_2_·6H_2_O per liter distilled water (Sidaway 1978)). After an aerobic incubation for 20 h, the calcifying solution was carefully removed. Then, 2.0 mL bacterial Mixture A and 0.5 mL bacterial Mixture B were added, and the tubes were incubated anaerobically at 37°C for 4 h. This procedure of replacing the calcifying solution with nutrient broth and vice versa was repeated for 14 days. The protocol was first checked by using glass slides in 24‐well plates. Calcification was determined by applying von Kossa staining (Figure 2). Another set of specimens was used for biofilm formation only. The pellicle was formed as described above. Then, bacterial Suspension A had been added for 8 h of incubation with 10% CO_2_. The media was removed and replaced with a mixture of bacterial Suspensions A and B in a 4:1 ratio. The tubes were then incubated in an anaerobic atmosphere for 16 h. Both the formation of biofilm only and the combined formation of biofilm and calculus are summarized in Figure 3.

### 2.3 Instrumentation

The instrumentation was performed by an experienced periodontist (G.J.), who was blinded to the group allocation (biofilm only or biofilm/calculus). According to the allocation (Figure 3), specimens were either instrumented manually with a Gracey curette (Deppeler SA, Rolle, Switzerland), referred to as “hand instrumentation,” or with a power‐driven ultrasonic scaler (AIRFLOW Prophylaxis Master, PIEZON PS (perio slim) instrument and PIEZON handpiece, Electro Medical Systems S.A., Nyon, Switzerland), referred to as “ultrasonic instrumentation.” Using the hand instrument, a series of five strokes was performed, and the specimen was subsequently dipped into a sterile 0.9% w/v NaCl solution to remove any detached bacteria. The ultrasonic group was instrumented at a set power level of 30% and irrigation level 70% with sterile water for 5 s, in accordance with the manufacturer's recommendation for the PS instrument.

### 2.4 Analysis of Biofilm and Calculus

The remaining biofilm was collected by intensive wiping with a sterile cotton swab and resuspended in 1 mL of 0.9% w/v NaCl. The suspension was intensively mixed and exposed to ultrasonication. Subsequently, serial dilutions were performed, and aliquots were plated on agar plates. After an incubation time of 8 days, the colony‐forming unit (cfu) was counted. The amount of calcium was determined using a commercially available kit (Calcium Assay Kit, Sigma‐Aldrich/Merck, Darmstadt, Germany) and the assay was performed according to the manufacturer's protocol. From each sample, 5 µL was transferred to wells of a 96‐well plate, and 200 µL working reagent was added. After incubation at room temperature for 3 min, OD was read at 620 nm.

### 2.5 Force Evaluation

The force sensor was calibrated before each instrumentation. The data acquisition during hand instrumentation depended on the time required for five strokes (mean: 3.7 ± 0.8 s). During ultrasonic instrumentation, 5 s of instrumentation were recorded. Pre‐ and post‐run times were recorded for all instrumentation. Concerning the forces to be analyzed, it must be considered that the handling of the two instruments differs: while in hand instrumentation a stroke is performed with a primary force vector in the *Z* direction (Coordinate system in Figure 1B), in ultrasonic instrumentation the instrument is moved gently along the dentin surface (*X*–*Z* plane) with a back‐and‐forth movement in the *X* direction, resulting in insignificant forces in the *Z* direction (Figure S1D,E). Therefore, the Fz forces, providing information about the resistance and adhesion of the substrate to be removed, were analyzed in detail only for hand instrumentation, and the Fy forces, associated with root substance defects, were analyzed and compared for both instrumentation groups. For this purpose, the maximum Fz values of the hand instrumentation data were evaluated and labeled (Figure S1A,B). However, during hand instrumentation, the instrument tip could get stuck on the edge of the cubic dentin specimen, leading to excessive forces. This issue was addressed by having the operator report all incidents of the instrument getting stuck and neglecting the corresponding force peak during post‐processing (e.g., third peak in H14_Bio in Figure S1A, label: excl.). The maximum force was replaced by the next highest Fz value, so that five peak forces were evaluated and compared for each hand instrumentation (Fz total *n* = 120). For the forces Fy, a single maximum value was identified for both hand (*n* = 24, Figure S1A,B) and ultrasonic instrumentation (*n* = 24, Figure S1D,E). Furthermore, the total force $F\;\text{total}\;=\;\sqrt{{\text{Fx}}^{2}+{\text{Fy}}^{2}+{\text{Fz}}^{2}}$ was calculated for all measurements (Figure S1A, B, D, E).

### 2.6 Statistical Analysis

All 12 independent biological samples per group, obtained in three independent experiments, were included in the statistical analysis. The calcium level (primary outcome) was recorded in pg/sample, the bacterial counts as log_10_ cfu, and the forces in N. When assuming a power of 80% and a significance level of 5%, 10 samples per group were required, when hypothesizing a standard deviation of 30% and a difference of 30% calcium level between the groups. Results were tested for normal distribution by applying Shapiro–Wilk test. Parametric data were compared by one‐way analysis of variance (ANOVA) with a post hoc comparison using Bonferroni correction. For the maximum peak forces Fy of all groups (except control), a Kruskal–Wallis test was performed, followed by a Mann–Whitney *U* test and Bonferroni correction. For the non‐normally distributed maximum peak forces Fz of both hand instrumentation groups, a Mann–Whitney *U* test was performed. A significance level of 5% was set for all statistical tests. The software SPSS 29.0 (IBM, Armonk, NY, USA) was used for all statistical tests.

## 3 Results

### 3.1 Biofilm, Calculus, and Removal

**Figure 4:**
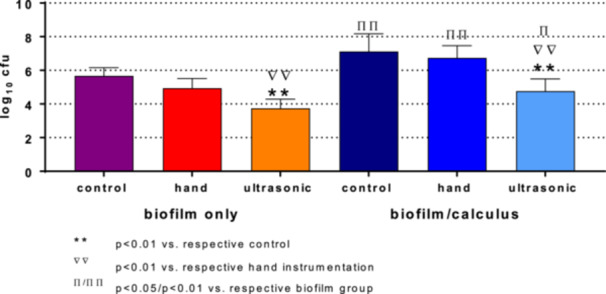
Number of colony‐forming unit (cfu) after hand and ultrasonic instrumentation of biofilm only and in combination with calculus.

The cfu counts in the biofilm/calculus groups were consistently higher than in the respective biofilm groups (Figure 4). The reduction using hand instrumentation was 0.73 log_10_ cfu in the biofilm and 0.38 log_10_ cfu in the biofilm/calculus group, without reaching statistical significance. For the ultrasonic instrumentation, the reductions were 1.92 log_10_ cfu (*p* < 0.001) in the biofilm only group and 2.36 log_10_ cfu (*p* < 0.001) in the biofilm/calculus group, respectively.

**Figure 5:**
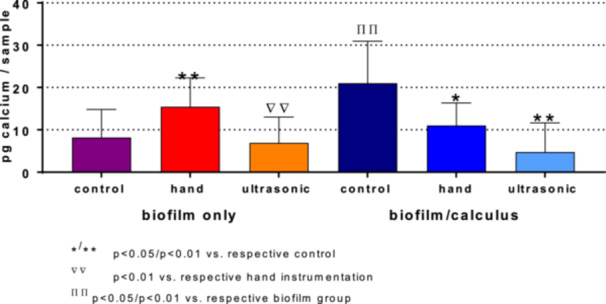
Calcium levels after hand and ultrasonic instrumentation of biofilm only and in combination with calculus.

The mean level of calcium, as a surrogate parameter for calculus, was significantly higher in the biofilm/calculus control group compared to the biofilm control (20 vs. 9 pg/sample, *p* = 0.002; Figure 5). Upon instrumentation, the calcium level increased after hand scaling in the biofilm only group (*p* = 0.009). In the biofilm/calculus groups, both hand and ultrasonic instrumentation decreased calcium levels (*p* = 0.029, *p* < 0.001). After both hand and ultrasonic instrumentation, the calcium level was not higher in the biofilm/calculus groups than in the biofilm only groups without calcification.

### 3.2 Force Evaluation

**Figure 6:**
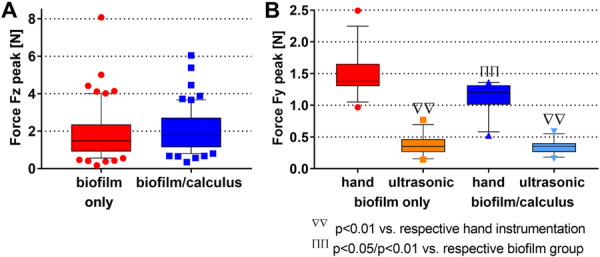
(A) Peak forces Fz (absolute values) during hand instrumentation of biofilm only and in combination with calculus; (B) peak forces Fy (absolute values) using hand and ultrasonic instrumentation of biofilm only and in combination with calculus.

Normality tests of the peak forces Fz of the hand instrumentation revealed a non‐normal distribution for both the biofilm and the combined biofilm/calculus groups (*n* = 60 each). Statistical analysis (Figure 6A) did not yield a significant difference between the two groups (*p* = 0.116), although a slightly higher median value was observed for the combined biofilm/calculus group (1.78 N vs. 1.48 N). The recorded force data are shown in Figure S1A,B, and a comparison of the detected and corrected peak forces Fz during hand instrumentation is provided in Figure S1C.

Statistical analysis (Figure 6B) of the peak forces Fy for all groups of the hand (Figure S1A,B) and ultrasonic instrumentation (Figure S1D,E) revealed that the peak forces Fy in the hand instrumentation groups were significantly higher for the biofilm only and biofilm/calculus groups (absolute medians: 1.37, 1.20 N), compared to the respective ultrasonic instrumentation (absolute medians: 0.35, 0.35 N; each *p* < 0.001). While the hand instrumentation groups showed a significant difference between biofilm only and biofilm/calculus (*p* = 0.005), no difference was found between the two ultrasonic groups.

## 4 Discussion

In the present study, a model was developed based on the formation of biofilm/calculus involving relevant microbial strains, enabling the assessment of periodontal instrumentation (in the present study, hand and ultrasonic instruments) with respect to biofilm and calculus removal, as well as the forces applied under laboratory conditions.

A series of experiments was conducted to establish an in vitro biofilm and calculus model that may reflect the subgingival region in periodontitis. Following this, a combination of anaerobic bacteria associated with periodontitis and calcifying species was used. Bacterial strains, including oral streptococci, *R. dentocariosa*, and *A. gerencseriae*, were selected and isolated from calculus samples as they were found to facilitate calcification. This aligns with other studies, which reported a potential for mineralization for oral streptococci (Karaaslan et al. 2020), *Rothia* spp., and *Actinomyces* spp. (Baris et al. 2017; Sidaway 1978). Oral streptococci were recently found to be predominantly in the saliva of rapid calculus developers (Cai et al. 2025). In the preliminary experiments, *P. gingivalis*, *T. forsythia*, and *F. nucleatum* were identified in the clinical calculus samples, which supports a very recent report about high numbers of these species in the calculus of periodontitis patients (Jin and Yip 2002). However, as others have observed (Karaaslan et al. 2020), we did not observe any calcification potential in the used reference strains *P. gingivalis*, *T. forsythia*, and *F. nucleatum*.

The challenge of the in vitro model was the combined formation of periodontal biofilm and calculus over an extended period of incubation. In preliminary tests, variables in the protocol were modified to increase biofilm mineralization. The highest mineralization was observed for pellicle formation with 0.5% BSA, the addition of 1 ppm sodium fluoride, pH 7.5 or 8.0 in the calcifying solution, and an intermediate aerobic incubation with repeated provision of viable bacteria. The increased albumin concentration at 1.5% BSA may negatively interfere with the precipitation of calcium phosphate (Wu et al. 2009). Sodium fluoride promotes precipitation (Jin and Yip 2002), therefore, 1 ppm sodium fluoride was added to the culture medium. Precipitation generally occurs at a higher pH. This was confirmed by an elevated mineralization when the calcifying solution was adjusted to pH 7.5 or 8.0. The advantage of the proposed protocol is a predefined biofilm composition that can be reproduced in a standardized way. Several in vitro studies have tested single species for their capability to form calculus (Karaaslan et al. 2020; Sidaway 1978), however, this is likely to reflect reality only to a very limited extent. In this study, only the total bacterial count was determined, rather than the count of individual bacteria, as qPCR or next‐generation sequencing would be required, which remains an interesting subject for future research.

A higher number of log_10_ cfu was found in the biofilm/calculus group compared to the biofilm only group, both before and after instrumentation. This was probably due to the extended incubation time and the initial calculus formation with increased bacterial adhesion. Treating the in vitro biofilm/calculus or biofilm only with hand or ultrasonic instrumentation reduced the number of log_10_ cfu counts, although statistical significance was only achieved with ultrasonic instrumentation. The reduction of less than 1 log_10_ cfu for the hand instrumentation (biofilm/calculus: −0.38 log_10_, biofilm only: −0.78 log_10_) may reflect clinical data, where the percentage of clean surface of single‐rooted teeth reached 62%–71% after treatment, depending on the depth of the periodontal pocket (Brayer et al. 1989). The reductions observed in the biofilm group after ultrasonic instrumentation were lower than those reported in literature, ranging from 2.85 to 4.08 log_10_ cfu (Berto et al. 2024; Hägi et al. 2015; Müller et al. 2011; Stähli et al. 2022). Possible reasons include the different biofilm model and a shorter treatment duration compared to other studies (Berto et al. 2024; Hägi et al. 2015; Müller et al. 2011; Stähli et al. 2022). In another study, the ultrasonic scaler was mounted, and the instrumentation was performed in a static modality. In this case, the reduction in cfu counts was lower despite a longer treatment time than in this study (Thurnheer et al. 2014). Data for the calculus/biofilm group are not comparable; to the best of the authors' knowledge, similar research has not been published until now.

The reduction of the calculus was validated by the calcium level of the residues after treatment. In the biofilm only group, the calcium level of the remaining biofilm increased after hand instrumentation. It can be assumed that the calcium originated from the dentin specimens. A recent in vitro study of our group found a higher hard substance loss when removing biofilm with a hand curette in comparison with an ultrasonic scaler (Hägi et al. 2015). However, in the present study, we did not quantify the surface damage and substance loss, which might be considered a limitation. After the instrumentation, the calcium levels of the biofilm/calculus groups decreased, and a substantial removal of the soft calculus could be assumed. However, these results did not show a significant difference compared to the respective biofilm only groups.

In addition, the mechanical properties such as hardness and adhesion of the in vitro calculus remain to be investigated and evaluated. Based on feedback during instrumentation, it can be concluded that the in vitro calculus is relatively soft with low adhesion to the dentin surface, comparable to newly formed dental calculus. A comparison with the results for ultrasonic instrumentation on an artificial soft dental calculus model (made of varnish and plaster mixture) using the same device and a comparable test setup by (Stutzer et al. 2024) revealed consistent findings: The absolute peak Fy values determined for the ultrasonic instrumentation of in vitro biofilm/calculus of 0.35 N ± 0.11 N corresponds well to the reported total median forces of 0.35 N ± 0.18 N and 0.28 ± 0.34 N. Furthermore, these values are also within the manufacturer's recommended lateral force limits of < 0.5 N (Hofmann et al. 2025). On the other hand, an in vivo study reported higher mean forces of up to 1.00 N during ultrasonic debridement on a section of a bicuspid (Ruppert et al. 2002). These increased forces could correlate to a greater calcification of the dental calculus. However, it should be noted that the force was measured on the handle of the ultrasonic scaler, and therefore likely represents the total force, unlike in the present study, where the force was measured on the substrate and thus provided information about the force direction. Moreover, Ruppert et al. concluded that the use of ultrasonic instruments may be highly subjective and that training is vital for effective use (Ruppert et al. 2002). An observation that is confirmed by Graetz et al. (2017).

The hand instrumentation of in vitro biofilm/calculus revealed higher lateral Fy forces than ultrasonic instrumentation (absolute medians: 1.20 N vs. 0.35 N), as expected, since theoretically no force needs to be applied by the operator during ultrasonic debridement. While hardly any difference was observed when using the ultrasonic scaler, a tendency towards higher forces was observed when applying hand instrumentation for biofilm only versus biofilm/calculus (absolute medians: 1.37 N vs. 1.20 N), albeit without statistical significance (*p* = 0.116). Although unexpected, this may underline the findings of higher calcium content in the group, as a potential indicator of greater tooth substance removal. No statistically significant statement can be made for the forces Fz during hand instrumentation. Furthermore, during hand instrumentation, it was observed that the instrument could occasionally become stuck at the edge of the dentin specimens, resulting in falsified, high Fz forces, which were subsequently excluded during post‐processing. For optimization, either larger specimens should be used, or the edge should be chamfered. However, compared to the literature, all valid Fz values of the hand instrumentation groups are within a reasonable force range, with a tendency to lower mean value: ranging from 0.18 to 8.1 N with a median of 1.60 N, compared to the reported range of 1.01 to 15.73 N with a mean of 4.62 or 5.28 N, depending on the operator group (Zappa et al. 1991). While the reference studies (Ruppert et al. 2002; Stutzer et al. 2024; Zappa et al. 1991) involved instrumentation performed by several dentists and dental hygienists, only one experienced periodontist was included in the present study. This was a conscious decision, which, in the context of minimal variation in device handling and in combination with a sample size of 12 per group, was intended to improve the study's validity. However, further studies should include multiple operators.

Clinically, there are no or minor differences in treatment outcome when instrumentation is performed with hand or ultrasonic instruments (Johnston et al. 2020; Malali et al. 2012; Obeid et al. 2004). Numerous clinicians use both hand and ultrasonic instruments, which can be advantageous in areas that are difficult to reach, such as furcations or deep defects. However, the overall treatment time might be shorter when using ultrasonic instrumentation only (Johnston et al. 2020; Kumari et al. 2023). When comparing both instrumentation modalities of the present study, the results were in favor of the ultrasonic scaler due to its superior removal of biofilm and calculus. In addition, both the lower lateral forces and lower calcium level in the biofilm only group are indicators of reduced damage to the dentin surface. However, this was not specifically verified by measuring changes in surface roughness and other in vitro studies report conflicting and inconsistent results regarding this conclusion. Kumari et al. (2023) found less surface damage on enamel and root cementum when using a hand instrument compared to an ultrasonic device. On the other hand, Graetz et al. (2017) applied different instrumentations to teeth coated with an artificial biofilm based on varnish. It was found that ultrasonic instrumentation resulted in significantly smoother surfaces compared to hand instrumentation, without any significant difference in biofilm removal. However, the comparability of the studies is limited. Although the same technological approaches, such as an ultrasonic scaler, were used, the specific devices, the design of the instrument tips, and the power settings used still have a considerable impact. The results of our study are therefore mainly supported by the consistent conclusions of the very similar study by Stutzer et al. (2024).

In summary, the developed model enabled an initial reproducible calculus formation and the evaluation of instrumentation, including the measurement of the forces applied. However, some limitations of the study need to be addressed. In vitro experiments can only fragmentarily represent clinical reality. A variety of factors are known to influence the amount and speed of calculus development, such as dietary sugars, salivary content and flow rate, age, ethnicity, gender, and bacterial load (Akcalı and Lang 2018). These factors could not be considered in the current in vitro model. To approach reality more closely, it might be interesting to generate calculus of a higher hardness by combining a resin coating as a base layer with the “biological” soft calculus. The sensor used allows determining the forces applied during the tested instrumentations, enabling to correlate the results with measurements of surface roughness and substance loss in subsequent studies. Together with a representative calculus model, the test setup developed could also be used to evaluate different types of instruments, including novel devices in the future.

## Author Contributions

S.E. and M.H. made substantial contributions to the conception and study design; G.J., L.G., and A.St. to the acquisition and analysis of data; and S.E. and M.H. to the interpretation of data. G.J. drafted the manuscript; L.G., A.St., A.Sc., J.B., S.E., and M.H. substantively revised it. All authors have approved the submitted version and have agreed both to be personally accountable for the author's own contributions and to ensure that questions related to the accuracy or integrity of any part of the work, even ones in which the author was not personally involved, are appropriately investigated, resolved, and the resolution documented in the literature.

## Conflicts of Interest

The authors declare no conflicts of interest.

## Supporting information

Supporting File

## Data Availability

The data that support the findings of this study are available from the corresponding author upon reasonable request.
