# Supplementary material for: Modeling In Vitro Biofilm–Calculus Formation for Assessing Periodontal Instrumentation and the Forces Applied
Source: Clin Exp Dent Res. 2026 Apr 22;12(2):e70359. doi: 10.1002/cre2.70359 (PMC13102088; doi:10.1002/cre2.70359)
Supplement: Supplementary file 1 — Supporting File [file CRE2-12-e70359-s001.pdf]

Supporting Information

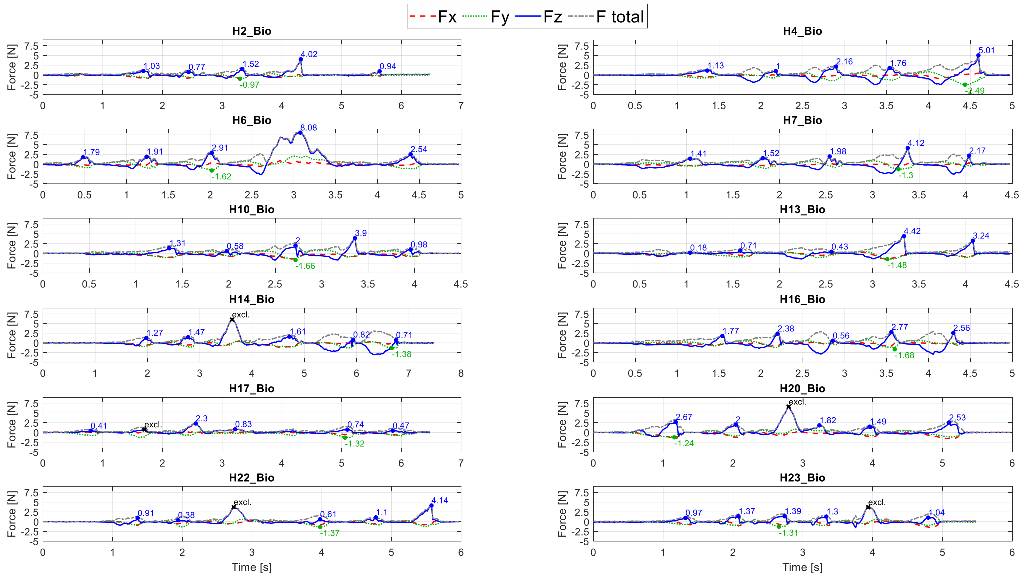

A

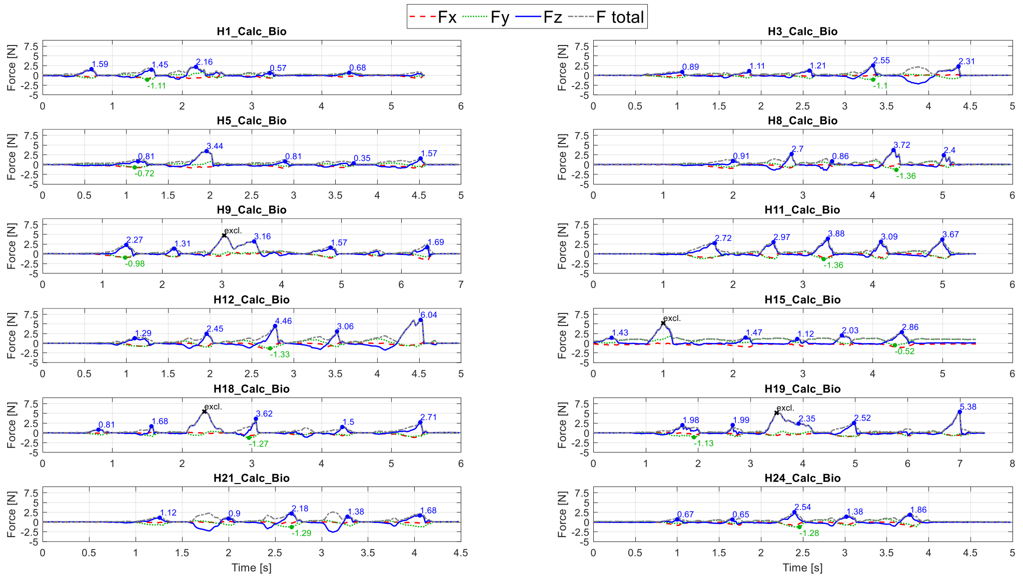

B

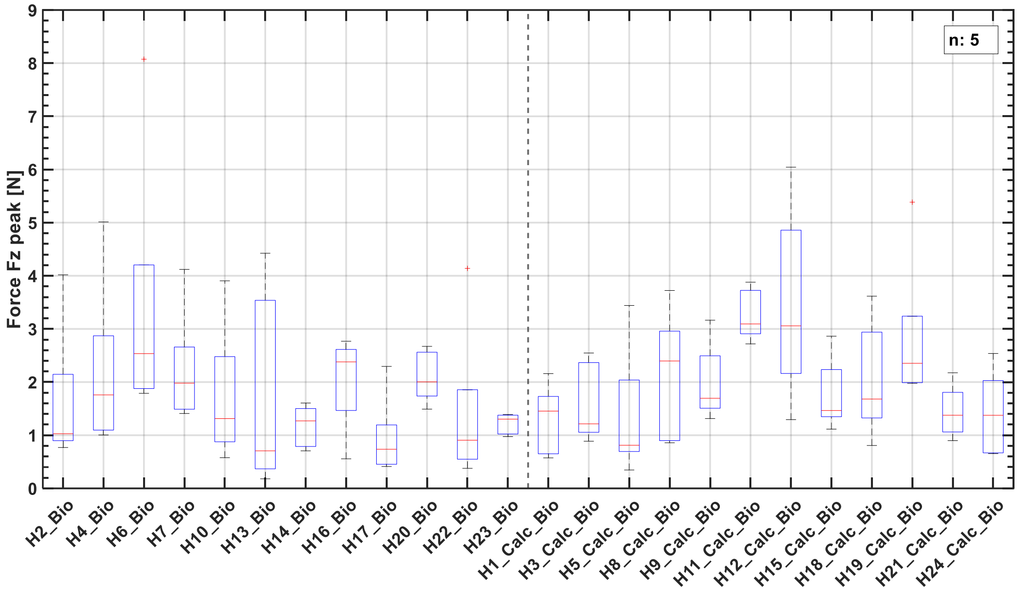
Force Fz peak [N]
n: 5
H2_Bio
H4_Bio
H6_Bio
H7_Bio
H10_Bio
H13_Bio
H14_Bio
H16_Bio
H17_Bio
H20_Bio
H22_Bio
H23_Bio
H1_Calc_Bio
H3_Calc_Bio
H5_Calc_Bio
H8_Calc_Bio
H9_Calc_Bio
H11_Calc_Bio
H12_Calc_Bio
H15_Calc_Bio
H18_Calc_Bio
H19_Calc_Bio
H21_Calc_Bio
H24_Calc_Bio

C

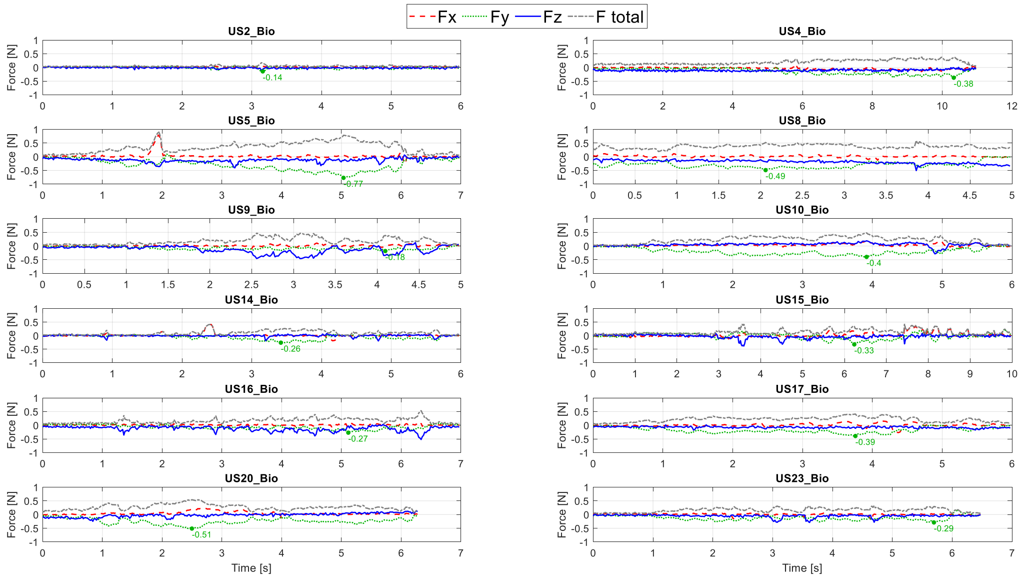
Fx
Fy
Fz
F total
US2_Bio
US4_Bio
US5_Bio
US8_Bio
US9_Bio
US10_Bio
US14_Bio
US15_Bio
US16_Bio
US17_Bio
US20_Bio
US23_Bio
Force [N]
Time [s]
-0.14
-0.38
-0.77
-0.49
-0.18
-0.4
-0.26
-0.33
-0.27
-0.39
-0.51
-0.29

D

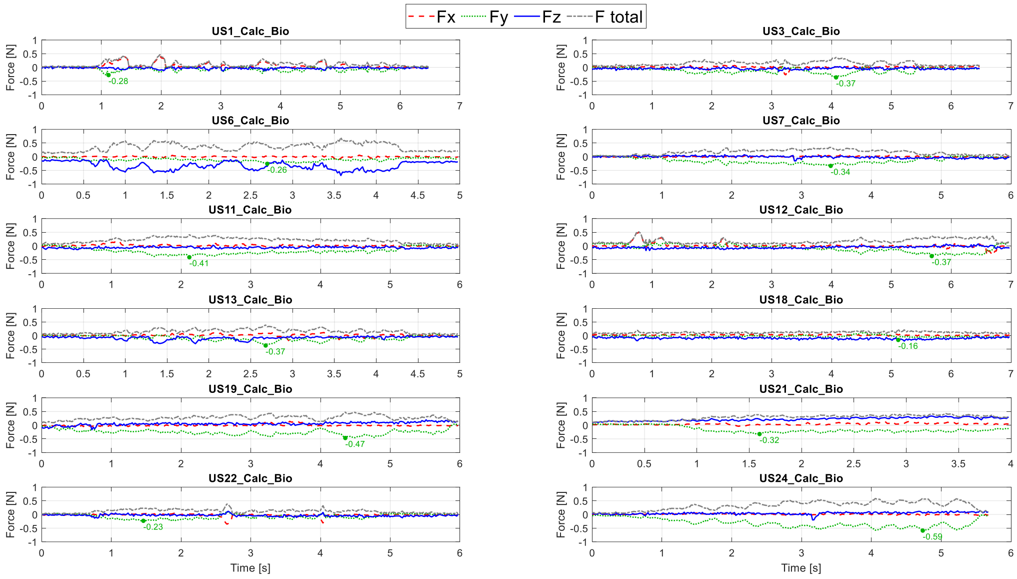

E

S1 Force measurement in X, Y, Z direction, peak indication and exclusions, and calculated total force for: (A) hand instrumentation of biofilm; (B) hand instrumentation of combined biofilm and calculus; (C) comparison of the five valid Fz peak values of all hand instrumentation; (D) ultrasonic instrumentation of biofilm; (E) ultrasonic instrumentation of combined biofilm and calculus.
